# Editorial: Psychosocial Interventions for Suicide Prevention

**DOI:** 10.3389/fpsyg.2019.02191

**Published:** 2019-09-24

**Authors:** Jorge Lopez-Castroman, Raffaella Calati

**Affiliations:** ^1^Department of Adult Psychiatry, Nimes University Hospital, Nimes, France; ^2^INSERM, University of Montpellier, Neuropsychiatry: Epidemiological and Clinical Research, Montpellier, France; ^3^Centro de Investigación Biomédica en Red de Salud Mental, Madrid, Spain; ^4^Department of Psychology, University of Milan-Bicocca, Milan, Italy

**Keywords:** suicide, prevention, caregivers, training, psychosocial risk assessment models

Psychosocial approaches to the understanding and prevention of suicidal behavior have been wished-for since the pioneer work of Emile Durkheim (nineteenth century), who studied suicide to demonstrate how social factors played a key role in the genesis of human behavior. Many years passed before his ideas took hold, but in the last few decades initiatives to reduce the rates of suicidal behavior are arising throughout the world. This Research Topic was conceived to provide a picture of current psychosocial approaches to prevent suicidal behavior. Our aim was attained thanks to a variety of remarkable contributions (11 articles) ranging from suicide survivors to social media coverage or medical training.

To start off, Cramer and Kapusta point out the necessity of articulating a multi-level approach to suicide prevention. The Social-Ecological Suicide Prevention Model contemplates risk and protective factors at four different levels: individual, interpersonal, community, and societal. The authors posit that such a model would help to build up an integrative theory of suicidal behavior and rationalize the application of suicide prevention policies. Their conclusions are linked to the perspective paper by O'Connor and Portzky that collected expert opinions to identify the key challenges and developments in suicide research and prevention. Far from been complacent, numerous major challenges are discussed. One of them concerns prevention through web and social media and it is treated very practically by Notredame et al. in another paper. Building on recent evidence, this paper outlines a “digitally augmented prevention policy” and describes three main functions of preventive actions through the web and social media: gatekeeper, communication outreach, and intervention outreach. Of course, applying such a model will imply a multidisciplinary effort and changes in our usual practice.

Some papers have focused on populations surrounding suicidal persons and deserving attention. This is especially the case of suicide survivors, who have rarely been described in detail despite their exposure both to a suicide model and to unusual levels of complicated grief. Bellini et al. provide a rare and detailed account on the mental health state of several suicide survivors participating in psychological autopsies. Because they might become suicide survivors, caregivers also need attention as important collaborators providing support in many different formats and being able to detect the transition from suicidal ideation to behavioral enaction. As pointed by Le Moal et al., few studies have focused on caregivers to improve suicide prevention. Medical students constitute another important population given their role in the community as future providers of care and access to care. Recent reports from different countries confirm that medical studies are commonly associated with elevated levels of stress, depressive symptoms and suicidal ideation. Gramaglia and Zeppegno discuss how medical training can enhance self-recognition of mental distress, reduce barriers to treatment-seeking and increase favorable attitudes toward suicidal behavior through personal experience. Finally, Brown et al. have studied the effect of a workshop for gatekeeper training in the attitudes, skill confidence and knowledge in a large sample of school staff. Although participation in this day and a half workshop induced a change in negative attitudes toward suicidal behaviors, the change did not last long. Participants, especially teachers, felt nonetheless more able to deal with suicidality even 6 months after the workshop.

Méndez-Bustos et al., undertook a systematic review of any observational paper investigating psychotherapeutic interventions to reduce suicide risk. These papers were not previously considered in prior meta-analytic studies and they provide interesting insights given the problematic adherence of suicidal patients to randomized controlled trials. The most relevant conclusions involve the need to increase the quality and exhaustiveness of reports in this domain and the interest of group and web therapies. The second systematic review and meta-analysis found that adolescents with self-harm were more engaged with treatment when they received any psychological therapy compared to treatment as usual (Yuan et al.) Treatment engagement was defined as attending four or more sessions and treatment as usual involved typical follow-up appointments with no structured therapy. This finding updates a previous meta-analysis that found no difference in engagement between the same groups, and supports the interest of structured psychological treatments in this population since non-adherence is rather the rule that the exception.

Interesting aspects of psychosocial interventions for suicide prevention are treated in mini-reviews. Prada et al. didactically describe the components of dialectical behavioral therapy (DBT) and how each of them has a precise effect in the reduction of self-harm and suicide attempts among patients with borderline personality disorder. The study of each module of a multi-component therapy, such as DBT, is the key to optimize future interventions. Since a one-fits-all treatment does not seem to be sufficiently effective in suicide prevention, we might need to design personalized treatments selecting the most convenient components. The second mini-review (Zeppegno et al.) contemplates psychosocial interventions for older adults. Not only the population of older adults is increasing very fast in developed countries, they are also more exposed that any other age group to the risk of suicide and more vulnerable to pharmacological treatments. However, only few studies had specifically addressed the issue of psychosocial interventions to this age group.

Following the lines traced by the contributions to the Research Topic, we can conclude that psychosocial interventions are effective but the challenges ahead are still important. Firstly, a personalization of treatment approaches depending on age, gender, or psychopathology is needed. Focusing future studies in the components of psychosocial interventions would help to determine which treatment program works better depending on the set of symptoms or the characteristics of a person. Finally, the advancement of the field requires innovation, notably through new computational methods (e.g., machine learning to predict treatment response), and new technologies (e.g., web-based or mobile phone–based tools), to encompass different levels of risk factors extending from the society to the individual in danger. In that quest, the role of the social environment that surround a suicidal person is not to be forgotten.

## Author Contributions

JL-C wrote the manuscript. JL-C and RC edited and revised the manuscript.

### Conflict of Interest

The authors declare that the research was conducted in the absence of any commercial or financial relationships that could be construed as a potential conflict of interest.

